# Insertion of Retrotransposons at Chromosome Ends: Adaptive Response to Chromosome Maintenance

**DOI:** 10.3389/fgene.2015.00358

**Published:** 2016-01-05

**Authors:** Geraldine Servant, Prescott L. Deininger

**Affiliations:** Tulane Cancer Center, Department of Epidemiology, School of Public Health and Tropical Medicine, Tulane University, New Orleans, LAUSA

**Keywords:** reverse transcriptase, telomerase, retrotransposons, target-site specificity, genome evolution, chromosome maintenance

## Abstract

The telomerase complex is a specialized reverse transcriptase (RT) that inserts tandem DNA arrays at the linear chromosome ends and contributes to the protection of the genetic information in eukaryotic genomes. Telomerases are phylogenetically related to retrotransposons, encoding also the RT activity required for the amplification of their sequences throughout the genome. Intriguingly the telomerase gene is lost from the *Drosophila* genome and tandem retrotransposons replace telomeric sequences at the chromosome extremities. This observation suggests the versatility of RT activity in counteracting the chromosome shortening associated with genome replication and that retrotransposons can provide this activity in case of a dysfunctional telomerase. In this review paper, we describe the major classes of retroelements present in eukaryotic genomes in order to point out the differences and similarities with the telomerase complex. In a second part, we discuss the insertion of retroelements at the ends of chromosomes as an adaptive response for dysfunctional telomeres.

## Introduction

**FIGURE 1:**
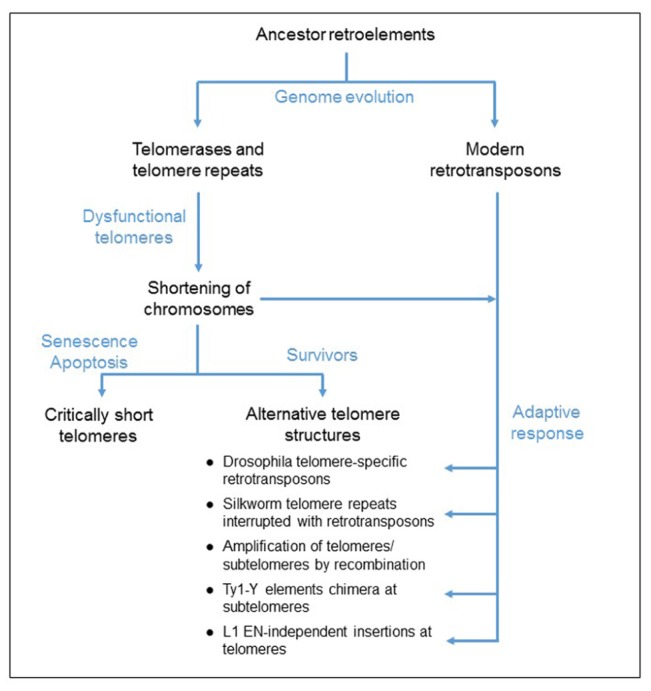
**Proposed model for the evolution of telomere elongation in eukaryotic genomes.** Both telomerases and retrotransposons derived from an ancestor retroelement. Mutations of the telomerase complex or protein-associated telomeres, inactivating the telomerase function, cause a shortening of telomeres. Critically short telomeres induce cell cycle arrest that can lead to cell death. Some cells survive to dysfunctional telomerase because of the formation of alternative telomere structures, generated by either homologous recombination mechanism or an adaptive response involving the activation of retrotransposition and *de novo* inserts at the chromosome ends.

In eukaryotic genomes, reverse transcriptase (RT) activity that leads to the synthesis of complementary DNA (cDNA) using an RNA template, is provided by two types of genetic elements, the telomerase gene and retroelements, also called retrotransposons. The telomerase reverse-transcribes a specific RNA template on to linear DNA ends to prevent the chromosome shortening caused by the replication mechanism (Blackburn, 1992). This is the first step of the formation of the complex nucleoprotein structures, the telomeres that cap and protect the chromosome ends (Muller, 1938; McClintock, 1941; Blackburn, 1992). Retrotransposons are mobile genetic elements that amplify their sequences throughout genomes, using an RNA intermediate and based on a “copy and paste" mechanism, termed retrotransposition (Boeke et al., 1985). Because these two genetic elements contain the same enzymatic activity and show some sequence similarity, it has been proposed that the telomerase complex has evolved from an ancestor retroelement and specialized to add nucleotides to the linear chromosome ends (**Figure 1**; Eickbush, 1997; Nakamura and Cech, 1998). The phylogenetic linkage between telomerases and retroelements has been reinforced by the identification of a group of retrotransposons, the Penelope-like elements, encoding a RT closely related to the telomerase enzyme (Arkhipova et al., 2003).

Retroelements have extensively colonized almost all eukaryotic organisms. For instance, 3% of the genome of the yeast *Saccharomyces cerevisiae* is made of retrotransposons (Kim et al., 1998). Retrotransposons also represent around 42, 37, and 3.6% of the genome of human, mouse and *Drosophila melanogaster*, respectively (Adams et al., 2000; Lander et al., 2001; Waterston et al., 2002). Because of their mobility and their high copy number, retrotransposons can generate gene disruption at the insertion site or cause genomic rearrangement by non-allelic homologous recombination. Therefore, they play an important role in the genome plasticity and they have a great impact on the architecture and evolution of eukaryotic genomes. In order for the elements to coexist with the cells, different strategies have been established to limit the damage caused by retrotransposition, including silencing of the elements (Hata and Sakaki, 1997; Bourc’His and Bestor, 2004) and destabilization of the new copies during the reverse transcription process by DNA repair proteins (Lee et al., 1998; Bryk et al., 2001; Gasior et al., 2008). A very efficient strategy to control the copy number in the genome is to direct the insertion in fairly safe regions, poor in genes, for example in heterochromatin or at telomeres (Okazaki et al., 1995; Zou et al., 1996; Takahashi et al., 1997).

Noteworthy in *Drosophila*, retrotransposons guarantee the protection of the chromosome ends because the telomerase is absent, probably lost during evolution (Biessmann et al., 1990). This observation suggests that RT activity is necessary to assure the function of protection of the linear chromosome ends and that retroelements could provide this activity in case of a dysfunctional telomerase. In fact, either activation of retrotransposition or integration of retroelements at telomeres has been reported in cells that survive a mutation in the telomere function (Scholes et al., 2003; Morrish et al., 2007). It has been proposed that this process is an adaptive mechanism to maintain the chromosome ends (**Figure 1**). In this review paper, we discuss the insertion of retrotransposons at telomeres.

## Retrotransposons and the Telomerase Complex

There are two major classes of retroelements: the long terminal repeat (LTR) retrotransposons, also called retrovirus-like elements, and the non-LTR retrotransposons. They are distinguishable based on structural features and the mechanism of retrotransposition.

### LTR-Retrotransposons

Long terminal repeat elements share similarities of structure and mechanism of replication with retroviruses. However, LTR-retrotransposons do not have a functional *env* gene, coding for a protein involved in cellular membrane recognition and cell invasion. Therefore LTR-retrotransposons are trapped in cells and are not able to escape or infect other cells. The best described elements are ZAM and Idefix of *Drosophila*, Ty retrotransposon in yeast *S. cerevisiae*, and IAP in mouse (for review Morgan et al., 1999; Prudhomme et al., 2005; Curcio et al., 2015; Mager and Stoye, 2015; Sandmeyer et al., 2015).

#### Structure

**FIGURE 2:**
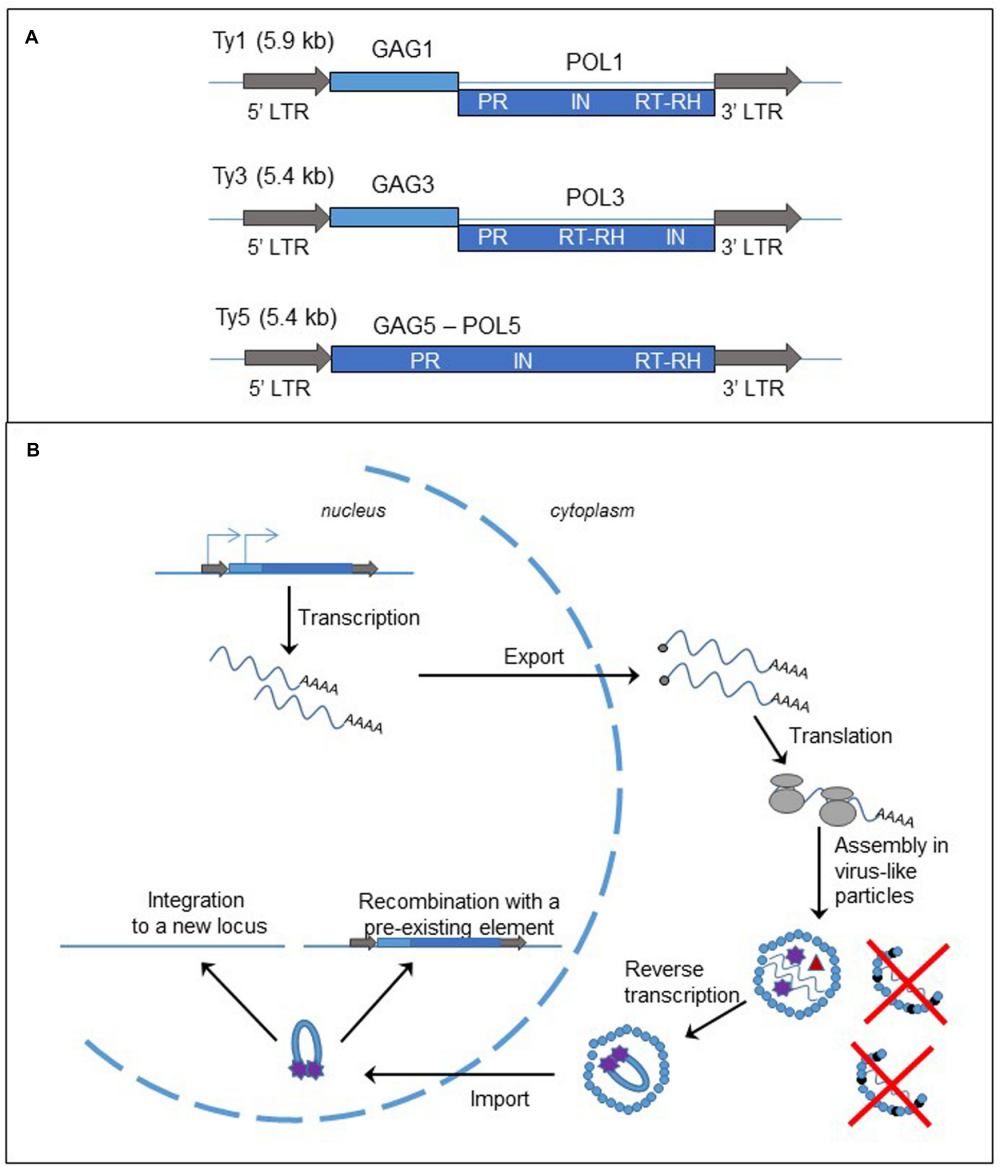
**Long terminal repeat retrotransposons, structure and replication cycle. (A)** Genomic organization of the yeast *Saccharomyces cerevisiae* retrotransposons, Ty1, Ty3, and Ty5. The gray arrows represent the LTRs; the light and dark blue boxes are the ORFs, GAG and POL, fused (Ty5) or separated by a frameshift (Ty1 and Ty3). LTR: long terminal repeat; PR, protease; IN, integrase; RT, reverse transcriptase; RH, RNase H. **(B)** Cycle of retrotransposition of LTR retrotransposons. The straight blue lines are the DNA strands. The light and dark blue boxes represent the two ORFs, GAG and POL, of the LTR-retrotransposon. The gray arrows are the LTRs flanking the two ORFs. The blue arrows on the left LTR and GAG represent the two initiation sites of the transcription of the element. The wavy blue lines represent mRNA of the element and the black dots at the left end is the cap. The gray circles are the ribosomes. The small blue circles represent GAGp and are organized in the VLP. The small black circles represent p22, the peptide responsible for Ty1 copy number control phenotype (destabilization of the VLPs). Inside the VLP the red triangle represent the reverse transcriptase, and the purple stars are the integrase.

Long terminal repeat-retrotransposons are flanked by LTRs, containing regulatory elements. These LTRs flank one or two open reading frames (ORFs), generally encoding GAG and POL proteins (**Figure 2A**). *GAG* and *POL* can be fused, as in the Ty5 element of *S. cerevisiae* (Zou et al., 1996; Neuveglise et al., 2002). Other LTR-retrotransposons contain two ORFs, either separated by a stop codon as in Tca2 of *Candida albicans* (Matthews et al., 1997; Neuveglise et al., 2002) or a frameshift as in Ty1 and Ty3 elements of *S. cerevisiae* (Clare et al., 1988; Neuveglise et al., 2002). As a consequence, both proteins are produced at different levels. GAG protein, the more abundant, is a structural protein that forms the virus-like particle (VLP). POL protein contains the protease (PR), RT associated with RNase H (RT/RH), and integrase (IN) activities. The organization of the domains in the POL protein is used for further classification of the LTR-elements in the two subfamilies, copia-Ty1 (PR-IN-RT/RH) and gypsy-Ty3 (PR-RT/RH-IN). LTRs possess the signals of initiation and termination of RNA polymerase (RNA pol) II transcription.

#### Retrotransposition Cycle

As described in **Figure 2B** and in several reviews (Curcio et al., 2015; Sandmeyer et al., 2015), the replication of LTR-retrotransposon starts with the transcription of a bicistronic RNA in the nucleus. The RNA is capped, polyadenylated and exported into the cytoplasm. Translation produces either a GAG protein or GAG-POL polyprotein. The polyprotein is processed by the protease encoded in the PR domain and the proteins are associated with two RNA molecules to form the VLP. A tRNA is also encapsulated in the VLP and serves as a primer for the synthesis of the cDNA. The reverse transcription occurs in the cytoplasm inside the VLP. Then the complex cDNA – integrase is imported into the nucleus. There are two mechanisms for the insertion of the new copy of retrotransposon to the genome. First the cDNA can be integrated to a new locus by the integrase activity. Second it can recombine with a pre-existing element through the homologous recombination process.

Ty1 retrotransposition and expression are controlled by Ty1 copy number (Jiang, 2002; Garfinkel et al., 2003) through an original mechanism that has been deciphered recently. The RNA interference pathway limits many retrotransposons, but the budding yeast does not have the machinery. Ty1 copy number is instead limited by a peptide, p22, expressed from a shorter and alternative Ty1 transcript and corresponding to the C-terminal domain of the GAG protein (Nishida et al., 2015; Saha et al., 2015; Tucker et al., 2015). The peptide interacts with the GAG protein, inhibiting its function, and destabilizes the VLP, leading to the decrease in the retrotransposition frequency and the alteration of stability or maturation of Ty1 proteins (**Figure 2B**).

#### Endogenous Retroviruses

The endogenous retroviruses (ERV) are also classified as LTR-retrotransposons. As the name suggests, they are remnants of ancient retroviruses that have infected the germinal cells of an ancestor organism and lost the ability to escape the cells. ERVs make up 8% of human genome but they are not currently active (Lander et al., 2001). Too many mutations have accumulated in their sequences, rendering the elements unable to retrotranspose. Some human ERVs can still express proteins and have a significant role in the cellular metabolism, such as the syncytin, a protein specifically expressed in placenta from a degenerated ERV and has an important role in the formation of the syncytiotrophoblast, a tissue that allow exchanges between the mother and the embryo (Heidmann et al., 2009; Lavialle et al., 2013).

### Non-LTR Retrotransposons

Non-LTR retrotransposons predominate in mammalian cells. In the human genome, the elements L1 and Alu are the most abundant and active mobile DNA species and constitute 17 and 11% of genome, respectively (Lander et al., 2001; de Koning et al., 2011). L1 is a long interspersed element (LINE) and encodes the activities required for its own retrotransposition. Alu element is a non-autonomous element, also called short interspersed element (SINE), and its replication relies on L1 protein expression.

**FIGURE 3:**
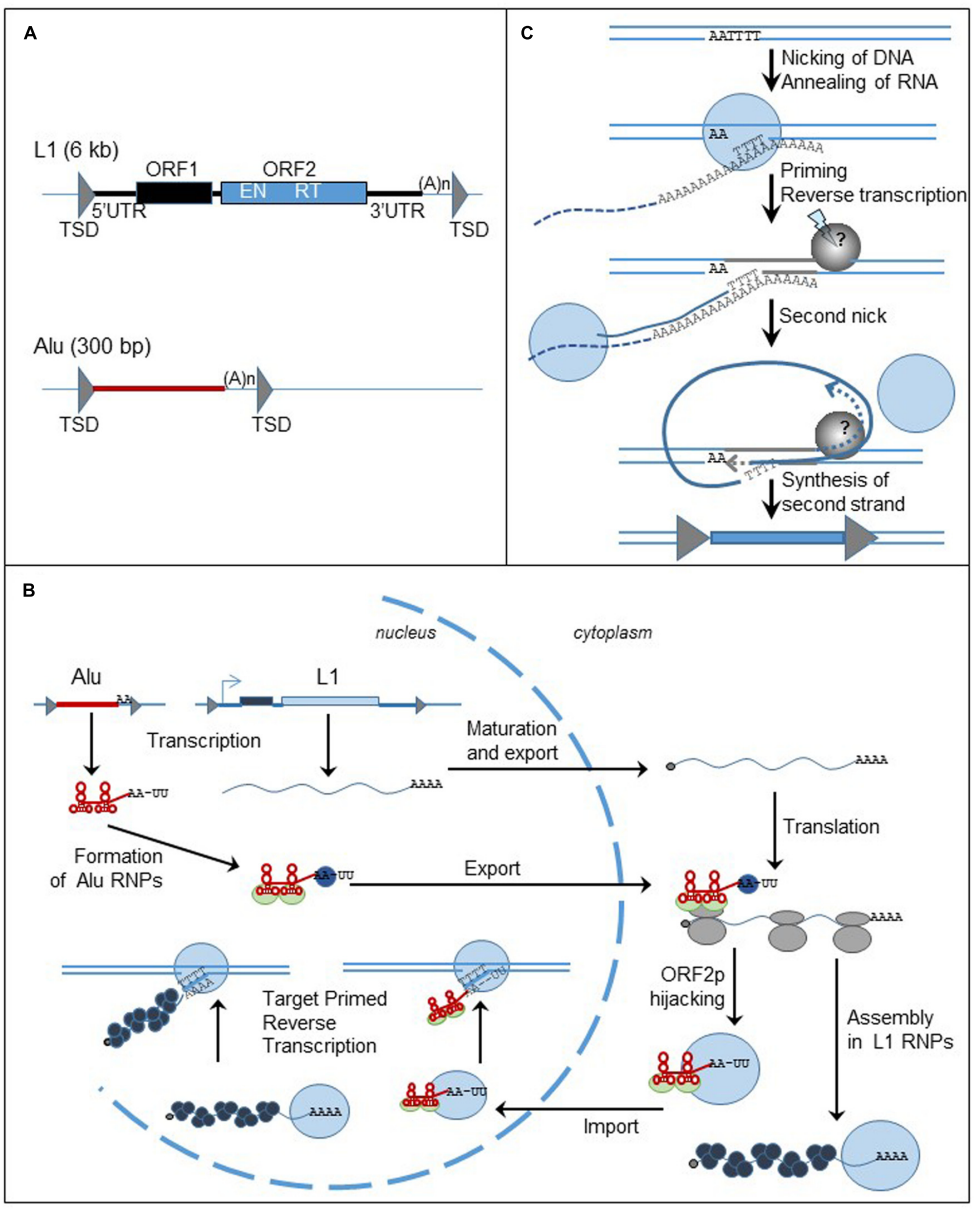
**Non-LTR retrotransposons, structure and replication cycle. (A)** Genomic organization of L1 and Alu elements. Triangles represent TSDs; black and blue boxed are the ORFs. UTR: untranslated region; TSD: target site duplication; ORF: open reading frame; (A)n: poly (A) tail; EN: endonuclease; RT: reverse transcriptase. **(B)** Cycle of retrotransposition of L1 and Alu elements. The straight lines are the DNA strands. Black and blue boxes represent ORF1 and ORF2 of L1 retrotransposon. The red box represent Alu element. The gray triangles flanking the boxes are the TSDs. The wavy blue lines represent L1 mRNA and the black dots at the left extremity is the cap. Alu RNA is represented by the red line. Attached to the red line, the light green circles are the SRP9/14 protein complex, the blue circles are PABP. The gray circles are the ribosomes. The blue circles represent ORF2p and the black circles represent ORF1p. **(C)** Mechanism of insertion of L1 element in the genome, the TPRT process. The lines are the DNA strands; the dashed lines are the RNA template. Blue circles represent ORF2p; the gray circle is the unknown protein responsible for the formation of the second nick. Gray triangles represent the TDS. The blue box represent the new insert.

Non-LTR retrotransposons represent a very broad group of retroelements, showing different features such as target-site specificity, enzymatic activities required for retrotransposition, or ORF number (Eickbush and Malik, 2002). In the present paper, we primarily focus on two model elements, the human L1 and Alu elements, in order to point out the differences with LTR-retrotransposons (**Figure 3**) and similarities and differences relative to the telomerase complex (for review Richardson et al., 2015).

#### Structure

The human genome contains about 500,000 copies of L1 elements (Lander et al., 2001). Out of them, only 6,000 are full-length, 6-kb long, and the others are generally 5′ truncated. L1 element consists of a 5′ untranslated region (UTR), two ORFs (ORF1 and ORF2), and a 3′UTR (**Figure 3A**). Inserts are flanked by target site duplications generated from the target site due to the mechanism of retrotransposition. ORF2 encodes the endonuclease (EN) and RT activities required to insert a new copy of the element to the genome (Mathias et al., 1991; Feng et al., 1996). In contrast, the function of ORF1 protein (ORF1p) is mostly unknown. However, ORF1p contains a nucleic acid binding domain, a chaperone activity, and a nucleolar localization signal (for review Martin, 2010). Both L1-encoded proteins are required for the mobility of autonomous elements (Moran et al., 1996). The L1 5′ UTR includes a RNA pol II promoter that assures the transcription of the element (Swergold, 1990; Severynse et al., 1992) and an antisense promoter (Speek, 2001). Recently, a third ORF, ORF0, has been discovered in the 5′ UTR of primate-specific L1 elements, expressed from an antisense promoter similar to the one previously described (Denli et al., 2015). The function of the protein still needs to be characterized but it seems that ORF0p modestly stimulates L1 retrotransposition. The L1 3′ UTR has a polyadenylation signal that is probably weak because some new L1 inserts include sequences from downstream of the original L1 elements (Moran et al., 1999). The process seems to be very frequent in cancer cells (Tubio et al., 2014). The L1 insert sequence ends with a poly (A) tail, a structure important for an efficient retrotransposition cycle (Moran et al., 1996; Doucet et al., 2015).

Alu elements, a 300 bp long, primate specific SINE, are related to 7SL RNA, the signal recognition particle (SRP) RNA (Quentin, 1992). They contain an internal promoter that allows them to be transcribed by the RNA pol III machinery. Alu inserts are flanked by TSDs and end with a poly (A) tail (**Figure 3A**). The presence of these structures, also important markers of L1 retrotransposition, supports the hypothesis that Alu elements share the same machinery as the L1 retrotransposon. However, enough differences in timing and factors influencing Alu retrotransposition, differentially from L1, indicate that their pathways diverge in many ways (Deininger and Batzer, 2002; Dewannieux et al., 2003; Wagstaff et al., 2013).

#### Retrotransposition Cycle

Based on the difference in the structure of the two groups of retroelements, it is not surprising that the elements do not share the same mechanism of retrotransposition. The main difference resides in the cellular location of the reverse transcription, occurring inside the VLP in the cytoplasm for LTR-retrotransposons and at the insertion site in the nucleus for LINEs and SINEs.

Briefly and as described in **Figure 3B** (for review Richardson et al., 2015), L1 mRNA, produced from the L1 promoter found within the 5′ UTR, is capped, polyadenylated and exported to the cytoplasm. L1 mRNA is translated into ORF1p and ORF2p as a bicistronic RNA. The proteins assemble with mRNA to form ribonucleoprotein (RNP) particles. It is not clear if the whole RNP is imported to the nucleus, but at least ORF2p and mRNA must enter into the nucleus. The reverse transcription of the mRNA occurs in the nucleus at the target site of insertion through a mechanism called target-primed reverse transcription (TPRT) (**Figure 3C**). The ORF2-EN domain recognizes and cleaves an AT-rich region. The T-rich DNA 3′ overhang anneals to the poly (A) tail of L1 mRNA and serves as a primer for the reverse transcription. The next steps of the mechanism are less characterized but a second nick is generated in order to finalize the insertion of the new copy of the element. The reverse transcription process can be interrupted before the synthesis of the full-length cDNA, generating a 5′ end-truncated element. Microhomologies with the genome are often found at the 5′ end of the truncated inserts suggesting that DNA repair machinery can disrupt the TPRT process (Zingler et al., 2005; Babushok et al., 2006).

The sequence analogy between Alu and 7SL RNA supports the hypothesis that Alu RNA can associate with the ribosomes. Similar to 7SL RNA, Alu RNA binds to the protein heterodimer SRP9/14, part of the SRP complex that binds to ribosomes and recognizes the signal peptide of secreted proteins during their translation (Hsu et al., 1995; Chang et al., 1996; Ahl et al., 2015). Therefore it has been proposed that the SRP9/14 complex could bring Alu RNA near the ribosomes and allow it to hijack L1 proteins during their synthesis (Dewannieux et al., 2003). Additionally, the length of the poly (A) stretch in Alu RNA is another important factor for the ability of Alu element to retrotranspose and it has been proposed that the poly (A) binding protein (PABP) may bind the poly (A) stretch and facilitate Alu RNA to associate with the translation machinery and then with L1 retrotransposition machinery (Roy-Engel et al., 2002; Dewannieux and Heidmann, 2005; Comeaux et al., 2009; Wagstaff et al., 2013). It seems that only ORF2p is really required for Alu mobility (Dewannieux et al., 2003), however, the presence of ORF1p seems to improve the efficiency of Alu retrotransposition (Wallace et al., 2008). Therefore L1 and Alu mobility are regulated differently.

### The Telomerase Complex, a Stringent Retrotransposon

**FIGURE 4:**
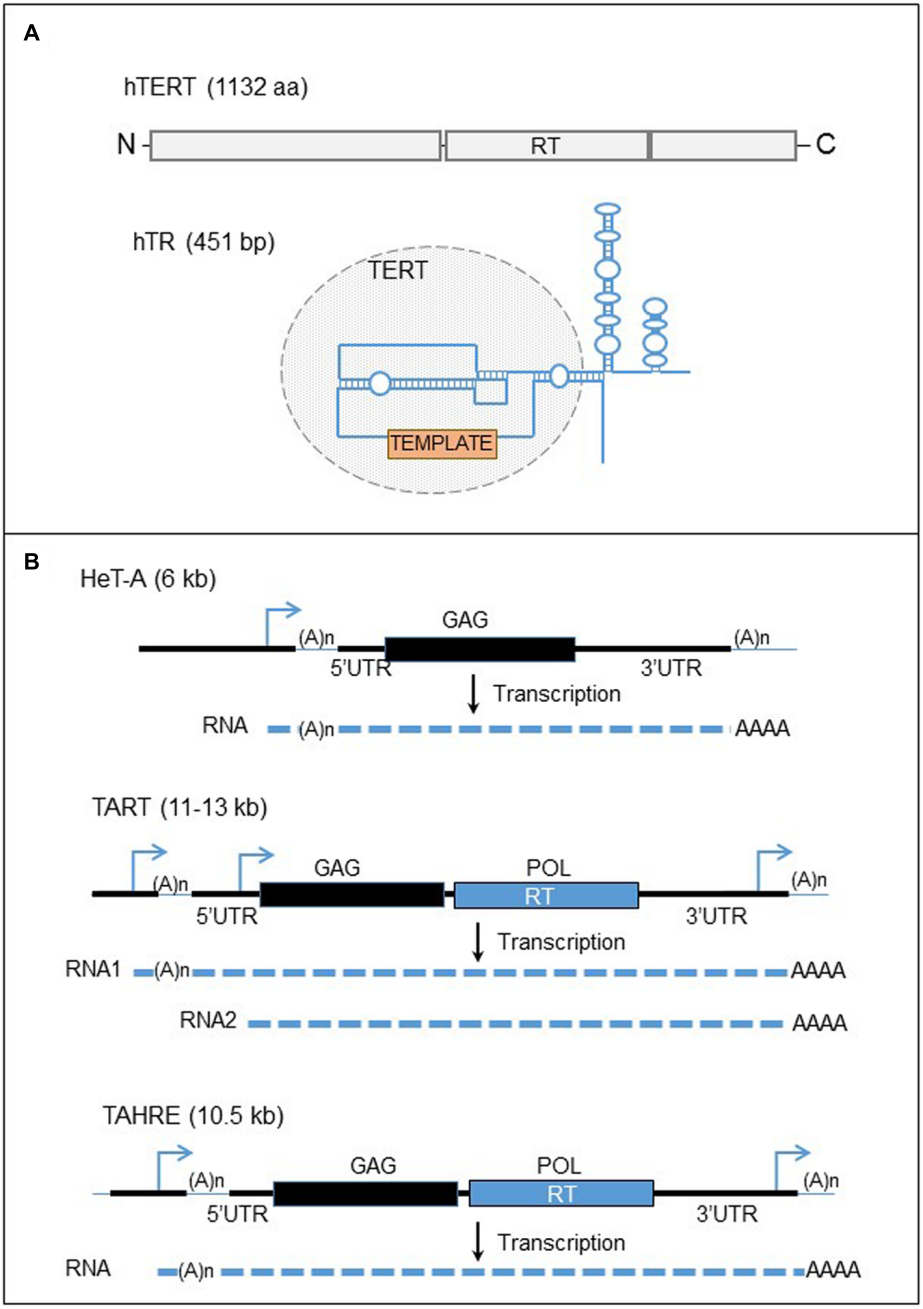
**Human telomerase complex and telomere-specific retrotransposons of *Drosophila*. (A)** The major components of the human telomerase complex. Top panel: organization of the human telomerase enzyme (hTERT). The gray boxes represent the three domains of the protein, the N-terminal, the reverse transcriptase (RT), and the C-terminal domains from left to right. Bottom panel: structure of the telomerase RNA template. The blue line represent the telomerase RNA. The circle domain represent the domain recognized by the hTERT. The orange box represents the template motif. **(B)** Telomere-specific non-LTR retrotransposons of *Drosophila*. Black lines are the DNA strands. The blue arrows represent the promoters of the elements. Black and blue boxes represent the two ORFs, GAG and POL. The dashed lines are RNAs. UTR, untranslated region; RT, reverse transcriptase.

The mechanism of telomere elongation is very similar to the non-autonomous, non-LTR retrotransposition process. In fact, the telomerase complex is organized in a complex RNP containing notably the telomerase (a RT enzyme), and a specific RNA template (**Figure 4A**; Greider and Blackburn, 1989; Feng et al., 1995; Harrington et al., 1997; Kilian et al., 1997; Lingner et al., 1997; Meyerson et al., 1997). The two components are located at two different loci in the genome and their expression is not linked. This system correlates with the RNP of a retrotransposon, constituted by a SINE RNA, such as human Alu RNA, associated with the LINE retrotransposition machinery. However, the two RNA templates are different. First the telomerase RNA template, including hTR in the human genome, is transcribed by the RNA pol II machinery and processed (Feng et al., 1995; Zaug et al., 1996; Mitchell et al., 1999). Second, the telomeric RNA template seems to be highly specialized, consisting in several domains necessary for both the assembly of the telomerase complex and notably catalytic activation of the telomerase: the telomerase binding domain, the template sequence for reverse transcription of telomere repeats, the telomerase-associated protein binding domains (for review Egan and Collins, 2012).

The telomerase protein, hTERT in human contains the RT activity. In contrast to RT encoded by retroelements, telomerase RT exists in one copy in the genome (Meyerson et al., 1997; Bryce et al., 2000). In addition, the enzyme does not bind and reverse transcribe its own mRNA with *cis* preference as the L1-ORF2p (Mitchell and Collins, 2000). In fact, the telomerase becomes active only after binding the telomerase RNA template and it has been identified that specific structures of the human RNA template are required for the catalytic activation of the enzyme (Mitchell and Collins, 2000). The telomerase complex assembles in the nucleus in Cajal bodies (Etheridge et al., 2002; Yang et al., 2002; Zhu et al., 2004; Venteicher et al., 2009). The two major components of the telomerase complex are associated with several proteins with multiple roles (for review Blackburn and Collins, 2011). The function of these proteins is really wide and diversified, and consists in the formation of the RNP, the regulation of telomerase activity, the regulation of the complex access to telomeres, and also the RNA stability, maturation and location.

The similarity between non-LTR retrotransposons and the telomerase complex is not only limited to the RNP structure because the reverse transcription of telomerase RNA template at chromosome ends utilizes a mechanism comparable to the TPRT process (Boeke, 1997; Eickbush, 1997), the insertion mechanism of non-LTR retrotransposon cDNA to the genome (Greider and Blackburn, 1989; Yu et al., 1990). However, in the case of the telomerase, the enzyme does not nick the DNA to prime the reverse transcription, but instead uses the 3′ OH end of the linear DNA to prime the reverse transcription. The RNA template is not entirely reverse transcribed at telomeres, only a small part of it, which also has some similarity to SINE TPRT. The elongation of telomeres is cell cycle dependent, and occurs during S-phase, when telomeres are uncapped and DNA is accessible (Jády et al., 2006; Tomlinson et al., 2006).

The role of the telomerase complex is essential for the maintenance of the genetic material because it allows for the synthesis of the chromosome extremities that the DNA polymerase is unable to amplify. Without this activity, replication would lead to chromosome shortening that could cause genome instability, senescence or apoptosis (Hayflick, 1979; Lundblad and Szostak, 1989; Harley et al., 1990; Levy et al., 1992). In humans, dysfunctional telomerase leads to diseases, such as dyskeratosis congenita, aplastic anemia, and pulmonary fibrosis (reviewed in Armanios and Blackburn, 2012). Alternatively, the length of the chromosome extremities are maintained through a mechanism of homologous recombination (for review Conomos et al., 2013). During the process, the 3′ OH end of the chromosome invades another chromosome end, and amplifies the repeats. Telomeres are thus dynamic structures and their sequence composition should be specific to prevent illegitimate recombination generating chromosomal rearrangements.

## Retrotransposition at the end of the Chromosomes: Specificity of Integration or Rescue of Dysfunctional Telomerase

### Telomere-Specific Retrotransposons

As a specialized retroelement, the telomerase complex targets specifically the chromosome extremities to reverse transcribe the RNA template. Interestingly, the telomerase complex is recruited to chromosome ends through specific interactions between telomerase enzyme and the shelterin complex, the telomere-associated proteins that cap the DNA ends (for review Nandakumar and Cech, 2013). In the fission yeast *Schizosaccharomyces pombe*, the phosphorylation of telomere capping proteins by the DNA damage sensor kinases, ATM and ATR, is required for the interaction with the telomerase complex and the recruitment at telomeres (Moser et al., 2011; Yamazaki et al., 2012). Such a regulation has not been yet characterized in mammalian cells but is suspected because ATM and ATR are also involved in telomere maintenance and notably telomere length regulation (for review Longhese, 2008; Diotti and Loayza, 2011). Intriguingly, important insights into the telomerase recruitment to chromosome ends were made by studying the mechanism of telomere healing, also called *de novo* telomere formation. Telomere healing is a very deleterious and rare process in the majority of eukaryote organisms that consists of adding telomere repeats at persisting DNA double strand breaks (DSBs) and leads to the loss of genetic information (for review Ribeyre and Shore, 2013). In budding yeast *S. cerevisiae*, telomere capping proteins and the telomerase complex are recruited to DSBs, in a comparable level as to telomeric ends, but the ATR ortholog, Mec1, limits their accumulation at DNA breaks and the *de novo* telomere formation (Zhang and Durocher, 2010; Ribaud et al., 2012). Therefore telomere healing can serve as a model to study the regulation of telomerase recruitment and activation in order to further determine the mechanism of protection of the linear DNA ends.

Retroelements have been identified and characterized in all sequenced eukaryotic genomes whereas they are a threat for the stability of the genomes. In human, their mobility, activated in germline cells, leads to diseases (for review Belancio et al., 2008; Hancks and Kazazian, 2012). The activity level of L1 elements is also very high but variable in a wide range of tumors (Iskow et al., 2010; Lee et al., 2012; Solyom et al., 2012; Tubio et al., 2014; Ewing et al., 2015). A very efficient way to prevent mobile DNA from generating gene mutations is to direct insertions in poor-gene regions. Subtelomeric and telomeric regions seem to represent a common “safe haven” for this purpose, although the multicopy rRNA cluster, and centromeric regions are used with some elements in some genomes. In this section, we examine the recruitment of telomere-specific retrotransposons, revealing similarities in the targeting mechanism of the telomerase complex, although the proteins involved may be different.

#### Target Specificity: Telomeres, Safe Harbor

The analysis of retrotransposons in genomes demonstrates that their distribution is not random and their location results of both integration specificity and selection pressure for the inserts that are less detrimental to the genome. The genome of *S. cerevisiae* is very condensed and retrotransposons are preferentially located in gene-poor regions of the chromosomes, either upstream of RNA pol III genes (Ty1, Ty2, Ty3) or at telomeres (Ty5) (Kim et al., 1998). In yeast, the integration bias is the consequence of a targeting strategy implying the interaction between the integrase and cellular factors, rather than the recognition of a specific DNA sequence by the enzyme.

In the genome of *S. cerevisiae*, there are few insertions of Ty5 retrotransposons and only one copy is full-length but not active because the coding regions contain several mutations (Voytas and Boeke, 1992). The inserts are located in the heterochromatin near telomere regions of chromosomes. Using an active Ty5 element from the related yeast strain *Saccharomyces parodoxus*, the Voytas laboratory has identified the mechanism of targeting specificity (Zou et al., 1996; Xie et al., 2001; Zhu et al., 2003). Ninety percent of *de novo* Ty5 elements are located in the silent chromatin at telomeres or silent mating loci and the integration is targeted through an interaction between the targeting domain of Ty5 integrase and the silent information regulator 4, Sir4p, a protein of the heterochromatin. Mutations in the targeting domain result in the loss of specificity of integration. Noteworthy, the integrase domain that interacts with Sir4p shares similarities with another protein interacting with Sir4p, Esc1p (Brady et al., 2008). Esc1p, a protein associated with the nuclear periphery, is also involved in chromatin silencing at telomeres (Andrulis et al., 2002). Additionally, the targeting domain is phosphorylated, and this post-translational modification mediates the interaction with Sir4p (Dai et al., 2007). The absence of phosphorylation results in a random integration of Ty5 elements in the genome and creates mutations. Intriguingly, the phosphorylation of integrase is regulated by stress conditions such as deprivation in nutrients (amino acids, nitrogen), suggesting that Ty5 retrotransposition is controlled for adaptive response to changes in environmental conditions.

Even if several copies of Ty1 retrotransposon of *S. cerevisiae* are recovered in subtelomeres, Ty1 is not a telomere-specific element. In fact, this location is a secondary target site selection and the targeting mechanism is not characterized. Ninety percent of Ty1 retrotransposons are preferentially targeted upstream of RNA pol III transcribed genes (Kim et al., 1998). The mechanism of this integration specificity has been recently identified and involves the interaction between Ty1 integrase and the cellular factor, AC40p, a subunit of RNA pol III complex (Bridier-Nahmias et al., 2015). When this interaction is lost, *de novo* Ty1 copies insert preferentially at chromosome ends. It has also been shown that the chromatin structure and chromatin remodeling complex are important components of the mechanism of the Ty1 integration upstream of RNA pol III transcribed genes (Bachman et al., 2005; Gelbart et al., 2005; Baller et al., 2012). Ty1 retrotransposons insert within 750 bases upstream of tRNA genes with a periodicity that depends on the nucleosome position in the region and more generally, Ty1 *de novo* inserts show a preference for nucleosome-rich sites, flanking RNA pol III transcribed genes (Baller et al., 2012). Therefore we can suppose that chromatin proteins can play a role in the insertion of Ty1 in heterochromatin at subtelomeric regions of chromosomes but the mechanism remains unknown and needs to be determined.

#### Retrotransposon to Compensate for the Absence of Telomerase in the Genome or a Low Expression Level of the Telomerase

The telomere-specific non-LTR retrotransposons of *Drosophila* represent an interesting case of domestication of transposable elements. The fly chromosome ends are not composed of canonical telomere repeats. The DNA component of the fly telomeres consists instead of three non-LTR retrotransposons arranged in tandem arrays, TAHRE, TART, and HeT-A (for review, Biessmann and Mason, 2003; Pardue et al., 2005; Pardue and Debaryshe, 2011; Fujiwara, 2015). Additionally, the genome of this organism does not encode a telomerase. The gene seems to have been lost in an ancestor of Diptera (Garavís et al., 2013). While some dipteran insects have maintained telomeric tandem repeats by homologous recombination, *Drosophila* genome has replaced the telomerase activity with the retrotransposition of the three telomere-specific retroelements. Therefore, RT activity from retrotransposons seems to be an adaptive cellular mechanism to recover a deficiency in the telomerase activity. Other *Drosophila* mobile elements are not found in the telomere arrays and the telomere-specific elements do not insert anywhere else in the genome, except for the broken ends of chromosomes (Biessmann et al., 1990; George et al., 2006).

The Pardue laboratory has described these elements and the telomere maintenance in *Drosophila* (**Figure 4B**). The sequence of the most abundant element, HeT-A, contains one ORF corresponding to a structure protein, ORF1, based on the domains present on the protein (Traverse and Pardue, 1988; Biessmann et al., 1990). Therefore HeT-A does not encode a RT activity and depends on another element for the retrotransposition. HeT-A is related to the latest discovered TAHRE element, encoding two ORFs (Abad et al., 2004). This element is less characterized because it is very rare at *Drosophila* telomeres. TART, the second most abundant element, has 2 ORFs and provide the retrotransposition machinery to the non-autonomous HeT-A (Sheen and Levis, 1994). Noteworthy, HeT-A ORF1p has a nuclear localization signal and the protein, fused to the green fluorescent protein (GFP), seems to form particles at chromosome ends in microscopy, whereas TART ORF1p does not have a specific cellular location (Rashkova et al., 2003). However, when the two proteins are overexpressed in *Drosophila* cells, both proteins co-localize at the end of chromosomes, suggesting that HeT-A ORF1p interacts with TART ORF1p and determines the intra-nuclear localization of TART proteins at the chromosome ends. The three non-LTR retroelements are assumed to insert specifically at the 3′ OH of the DNA end at the chromosome extremities. Therefore, an EN activity is dispensable for a retrotransposition event to occur. The promoter of HeT-A elements is in the 3’UTR whereas several promoters are located at both ends of the TART element (Danilevskaya et al., 1997, 1999). Therefore the transcription of an element can start from the 3′ end of the last element inserted at the end of chromosome, an apparent adaptation to retroelements appearing in tandem arrays.

Because *Drosophila* does not have canonical telomere repeats and telomerase complex, it is not surprising that proteins capping chromosome ends, constituting the terminin complex, are original and do not have sequence homology with proteins in human and yeasts (review Raffa et al., 2011, 2013). However, the function of terminin proteins such as HOAP and HipHop, is conserved: they are recruited to chromosome ends, accumulate, and prevent the action of DNA repair pathways on the chromosome extremities (Rashkova et al., 2002; Gao et al., 2010). The regulation of the recruitment of these proteins to telomeres is also conserved and involves the DNA sensor kinases ATM and ATR, which also regulate the formation and maintenance of telomeres in the other organisms (Bi et al., 2005; Gao et al., 2010). The mechanism of recruitment of terminin proteins to chromosome ends is unknown and the interaction with the proteins of telomere-specific retrotransposons has never been characterized. Interestingly, the understanding of telomere maintenance in *Drosophila* has also benefited from studies of DSB repair by telomere healing. Actually chromosomes lacking telomere-specific retrotransposons are remarkably stable for several generations, even in natural fly populations (Biessmann et al., 1992; Ahmad and Golic, 1998; Kern and Begun, 2008). Additionally, while the process of *de novo* telomere addition involves the RT activity of the telomerase complex in most organisms, surprisingly the establishment of *Drosophila* caps at DNA ends does not require the retrotransposition of telomere-specific elements for the assembly and maintenance of a functional terminin complex (Gao et al., 2010; Beaucher et al., 2012). Therefore, even if the loss of telomerase complex in evolution changed the proteins involved in chromosome end cap, the function and mechanism of maintenance are conserved.

The silkworm, *Bombyx mori*, appears to be a hybrid of canonical telomeres with retrotransposon-based telomeres (for review Fujiwara, 2015). In this case, the telomere repeats are interrupted with two families of non-LTR retrotransposons, SART and TRAS (Okazaki et al., 1995; Takahashi et al., 1997). The telomerase activity in this organism is barely detectable and to maintain the length of the chromosome extremities, these autonomous retroelements target specifically the telomere repeats (Sasaki and Fujiwara, 2000). Intriguingly only full-length elements are identified at telomeres (Fujiwara et al., 2005). Some copies have been reported in other part of the chromosomes, mostly truncated and not at the target site (Monti et al., 2013). They may be the result of recombination events between elements at telomeres and sequences in the genome. SART and TRAS elements have a very similar structure to human L1 retrotransposons. They encode two ORFs, ORF1 and ORF2 (Okazaki et al., 1995; Takahashi et al., 1997). ORF2p has EN and RT activities. The EN domain recognizes the telomeric repeats, TTAGG, and cleaves specifically between T and A. TRAS ORF1p has a nuclear localization domain and is able to interact with ORF2p (Matsumoto et al., 2004). However, the specific role of ORF1p is not well understood. Both proteins are required for the mobility of SART and TRAS. Unlike L1, the 3′ UTR of the silkworm telomere-specific elements is also required for retrotransposition (Takahashi and Fujiwara, 2002). The 3′ UTR has specific motifs that are proposed to interact with the RT domain of ORF2p and to anneal to the target site (Osanai et al., 2004). Although these non-LTR retrotransposons are actively transcribed, promoter motifs have not been identified (Takahashi and Fujiwara, 1999). The activities of the telomerase complex and the telomere-specific retrotransposon may be in conflict if they occur at the same time. However, while the telomerase complex is regulated by the cell cycle, such a regulation has not been reported for SART and TRAS retrotransposons in *Bombyx mori*. Additionally little is known about the mechanism of the recruitment of these elements to the telomeric repeats and it is possible that cellular factors may direct the recognition of the target sequence by ORF2p.

### Redirection of the Insertion in Case of Deficient in Telomere Maintenance: Impact on Genome Stability

It is intriguing to note that some telomere-specific retrotransposons seem to rescue partial or complete deficiencies of the telomerase activity. This observation suggests that retrotransposition may serve as a response to dysfunctional telomerases or to the absence of telomerase in cells.

The Curcio laboratory has studied the regulation of Ty1 retrotransposition in yeast strains defective in telomerase. In yeast, the telomere RT Est2p uses RNA template Tlc1 to polymerize telomere arrays at the chromosome extremities (for review Lundblad, 2002; Kupiec, 2014). In yeast strains deficient for the telomerase activity, the *est2*Δ mutants, telomere length decreases with cell divisions until the telomere length becomes very short and causes the arrest of cell division (Lundblad and Szostak, 1989). Usually cells stop dividing after 50 to 100 generations. Rare cells survive and present alternative telomere structures (Lundblad and Blackburn, 1993). Type I survivors contain tandem arrays of subtelomeric repeat Y’ and type II survivors have long and heterogeneous tracts of telomeric repeats. Scholes et al. (2003) has reported that Ty1 retrotransposition is induced in the *est2*Δ mutant, before cell senescence and the appearance of survivors. The activation of Ty1 retrotransposition frequency occurs in parallel with telomere erosion and is characterized by an increase in Ty1 cDNA in cells. However, in survivors, the Ty1 retrotransposition rate decreases. Therefore Ty1 retrotransposition is induced as a response to telomere dysfunction and raise the question whether this activation plays a role in the formation of alternative telomeres. In another publication, the Curcio laboratory showed that chimeric Y’-Ty1 elements are identified in type I survivors (Maxwell et al., 2004). Ty1 retrotransposon contributes to the retrotransposition of the Y’ repeats at subtelomeres in telomerase-deficient cells. Retrotransposition seems to be, in this case, one mechanism allowing for the extension of telomeres in telomerase-negative survivors. Intriguing the authors also showed that Y’ RNA is enriched in Ty1 VLP fraction and that this enrichment is not regulated by telomere erosion because Y’ RNA is present in the VLPs of telomerase-positive and negative cells. These data suggest that the integration events of Y’ cDNA only occur in telomerase-deficient cells and raise the question of which cellular factors are involved in this control.

In contrast, L1 retrotransposition has not been reported to be activated in cells deficient in telomerase activity. However, there are EN-independent L1 events that have been reported to be inserted at the chromosome extremities. EN-independent events have been first characterized in the Moran laboratory, looking at the effect of the deficiency in the non-homologous end joining (NHEJ) DSB repair in mammalian cells (Morrish et al., 2002). They identified that normal L1 retrotransposition is not noticeably induced in this mutant, but they observed unusual events, that lack common marks of L1 retrotransposition such as TSDs, or common EN target site at the insertions. Additionally the *de novo* L1 copies are 3′ end truncated, suggesting that these insertions have occurred at DNA lesions. In DNA PKcs-deficient cells, 30% of L1 EN-independent retrotransposition events have occurred at telomeres (Morrish et al., 2007). These events are not observed in another cell line deficient for XRCC4, an essential component of the NHEJ pathway (reviewed in Williams et al., 2014). DNA PKcs is very well identified as an essential kinase of the NHEJ pathway (for review Lees-Miller and Meek, 2003; Weterings and Van Gent, 2004). More recently, DNA PKcs has been reported as a component of the telomere maintenance. In fact, cells mutated in the kinase have uncapped dysfunctional telomeres, but unaffected in their length (Goytisolo et al., 2001; Williams et al., 2009). Morrish et al. (2007) showed that the new L1 inserts at the telomeres in DNA PKcs mutant can exhibit a poly (A) tail but the retrotransposition did not occur at common EN target sites. These observations imply that uncapped dysfunctional telomeres, but not shortened telomeres, are substrates for opportunistic L1 RT in mammalian cells. These data suggest that the L1 retrotransposition machinery is recruited to unprotected and persistent DNA ends and this phenomenon resembles the process described as *de novo* telomere formation at DSBs by the telomerase complex. Intriguingly, L1 retrotransposition at the chromosome ends, in this study, does not supply the absence of telomerase activity, revealing a more general response of the retrotransposons to the dysfunction of telomere maintenance.

## Conclusion

Telomerases have likely evolved from an ancestor retroelement during genome evolution (**Figure 1**). They are essentially stringent non-autonomous retrotransposons, specialized to insert telomeric repeats at the linear chromosome ends. The description of telomerases and modern retrotransposons reveals the specificities of each group of genetic elements. Notably, the originality of the telomerase RT function is based on the exclusivity of the RNA template and this is a very unique mechanism of regulation. In fact, although retrotransposon enzymes preferentially bind and reverse transcribe their own encoding RNAs, they are able to recognize other RNAs. Therefore, they are responsible for the insertion of processed pseudogenes throughout the genome, and also they supply the machinery to amplify non-autonomous retroelements (Derr et al., 1991; Esnault et al., 2000; Wei et al., 2001). In contrast, telomerase complexes cannot reverse transcribe other sequences in the genome because the presence of the specific RNA template in the active site of the enzyme is necessary for the catalytic activation. Therefore the telomerase complexes are very unique genetic elements in eukaryotic genomes and mutations disrupting the telomerase function cause the shortening of telomeres and the arrest of the cell cycle. Telomerase-negative survivors need to develop alternative pathways to compensate for the shortening of the chromosome ends. We discussed in the present paper the possibility that retrotransposition might provide an adaptive mechanism for the formation of alternative telomere structures and compensate for the shortening of the chromosomes (**Figure 1**).

Two examples especially seem to validate this hypothesis: the *Drosophila* and silkworm telomere-specific non-LTR retrotransposons. These retrotransposons are specialized and are not inserted anywhere else in the genomes. Furthermore, the chromosome extremities of *Drosophila* and silkworm are also protected from the integration of other retrotransposons that are not telomere-specific. Because telomerase complexes are phylogenetically closer to non-LTR retrotransposons, notably based on the similarity of the insertion process, it is easy to imagine that non-LTR retrotransposons can counteract the shortening of the chromosomes in cells deficient for the telomerase function. However, in response to disrupted telomerase gene, the budding yeast *S. cerevisiae*, containing only LTR-retrotransposons, activates Ty1 RT, contributing to the formation of alternative telomere structures in survivor cells. Therefore, retrotransposition seems to be an evolutionary mechanism to compensate the telomerase deficiency. Intriguingly the comparison of the different mechanisms of chromosome end protection also reveals similarities in the recruitment of the telomerase complex and retrotransposons to the target sites, providing new perspectives for the investigation of telomere formation and maintenance.

## Author Contributions

GS and PD wrote the paper.

## Conflict of Interest Statement

The authors declare that the research was conducted in the absence of any commercial or financial relationships that could be construed as a potential conflict of interest.
